# Promising Roles of Circular RNAs as Biomarkers and Targets for Potential Diagnosis and Therapy of Tuberculosis

**DOI:** 10.3390/biom12091235

**Published:** 2022-09-04

**Authors:** Yifan Huang, Ying Li, Wensen Lin, Shuhao Fan, Haorong Chen, Jiaojiao Xia, Jiang Pi, Jun-Fa Xu

**Affiliations:** 1 Guangdong Provincial Key Laboratory of Medical Molecular Diagnostics, The First Dongguan Affiliated Hospital, Guangdong Medical University, Dongguan 523808, China; 2 Institute of Laboratory Medicine, School of Medical Technology, Guangdong Medical University, Dongguan 523808, China

**Keywords:** circular RNAs, tuberculosis, biomarkers, immunity, pathology

## Abstract

Tuberculosis (TB), caused by *Mycobacterium tuberculosis* (Mtb) infection, remains one of the most threatening infectious diseases worldwide. A series of challenges still exist for TB prevention, diagnosis and treatment, which therefore require more attempts to clarify the pathological and immunological mechanisms in the development and progression of TB. Circular RNAs (circRNAs) are a large class of non-coding RNA, mostly expressed in eukaryotic cells, which are generated by the spliceosome through the back-splicing of linear RNAs. Accumulating studies have identified that circRNAs are widely involved in a variety of physiological and pathological processes, acting as the sponges or decoys for microRNAs and proteins, scaffold platforms for proteins, modulators for transcription and special templates for translation. Due to the stable and widely spread characteristics of circRNAs, they are expected to serve as promising prognostic/diagnostic biomarkers and therapeutic targets for diseases. In this review, we briefly describe the biogenesis, classification, detection technology and functions of circRNAs, and, in particular, outline the dynamic, and sometimes aberrant changes of circRNAs in TB. Moreover, we further summarize the recent progress of research linking circRNAs to TB-related pathogenetic processes, as well as the potential roles of circRNAs as diagnostic biomarkers and miRNAs sponges in the case of Mtb infection, which is expected to enhance our understanding of TB and provide some novel ideas about how to overcome the challenges associated TB in the future.

## 1. Introduction

Tuberculosis (TB) is one of the most common infectious diseases in the world, predominantly caused by one of the most tricky pathogens, *Mycobacterium tuberculosis* (Mtb), which lies dormant in the host cells by escaping the host immune clearance [1,2]. Among people infected with Mtb, totaling a quarter of the population worldwide, 5–10% of the infected patients will develop active TB disease (ATB) [1]. To the present time, the increasing emergence of multidrug resistant TB has been a major barrier to public health security, reported to be responsible for over ten thousand deaths related to antimicrobial resistance [2]. The co-infection conditions, such as human immunodeficiency virus (HIV) co-infected TB, and other disease coexisted conditions, such as diabetes coexisted TB, make the pathogenesis of TB more a more complicated question as to cure and control of the disease [3,4]. Other factors, such as the widespread prevalence of the tuberculosis bacterium of high virulence [5] and uncontrollable relapse TB cases [6,7], also present challenges to TB disease control. Currently, the only licensed tuberculosis vaccine, Bacillus Calmette-Guérin (BCG) is recommended at birth to protect children against tubercular meningitis, but its protective effects against TB are very poor in adults [8,9]. Moreover, Mtb can remain dormant in the host cells for a long time [10], which introduces an asymptomatic latent TB infection condition, likely detectable by an interferon-γ release assay, but without other combined methods for accurate and rapid diagnosis. Therefore, it is urgent to clarify the pathological and immunological mechanisms in the development and progression of TB, an effort which might benefit the developments of more effective strategies for TB prevention, diagnosis and treatment.

Circular RNAs (circRNAs) are a kind of non-coding protein RNA expressed in most eukaryotic cells. Compared with linear RNAs, the absence of five-prime caps and three-prime tails allow circRNAs to loop into a single-stranded, covalently closed structure for more stable and highly conserved characteristics, which helps to avoid or escape from RNAse digestion [11,12]. In the 1970s, viroids in plants, exhibiting high thermal stability and single-stranded covalently closed structure, were found to be the first circular RNA by Sanger et al. [13], which further confirmed by sequence analysis two years later [14]. This work thus opened up a new field of biology and medicine for the functional and mechanistic study of circRNAs in different species.

In the 1990s, researchers serendipitously discovered that the products of splicing transcripts with an inverted order of exons, called mis-splicing, which occurred specifically at consensus splice sites, could lead to the formation of circular RNA molecules, such as the tumor suppressor gene deleted in colorectal carcinomas (DCC) [15] and human proto-oncogene *ets-1* [16]. These non-polyadenylated scrambled transcripts were found at much lower levels than in the normal transcripts [17]. However, the prevalence and the significance of circRNAs were not discovered over the ensuing decades, until high-throughput sequencing could be applied. With the assistance of deep sequencing, substantial circRNAs were discovered and were no longer simply considered to be accidental byproducts of splicing [18]. Salzman et al. identified numerous RNA isoforms with scrambled exons in normal and malignant human cells, and then confirmed that these scrambled-exon transcripts were circRNAs enriched in cytoplasm. These results proved that circRNAs were ubiquitous, and that most of them were highly expressed in human cells [11]. After this discovery, circRNAs in human cells began to receive extensive attention, and the emerging functions of circRNAs were subsequently identified.

In this review, we systematically summarize the characteristics, biogenesis, detection methods and biological functions of circRNAs. Moreover, we further discuss the critical roles of circRNAs in the development and progression of TB, by which we believe that one could enhance our understanding of the pathological and immunological mechanisms of TB at genetic level and finally benefit by achieving a more effective control of TB.

## 2. The Biogenesis of circRNAs

CircRNAs are generated from precursor mRNAs by head-to-tail back-splicing [19]. Previous research has indicated that the biogenesis and circularization of circRNAs can be influenced by *cis*-regulatory elements [20], *trans*-acting splicing factors [21], or a combination of both [22,23]. Inverted repeated Alu elements (IRAlus) are located in the intronic flanks of circularized exons for RNA editing [20], while *trans*-acting splicing factors such as quaking (QKI) [24] exist in special tissues for specific controls of splice site selection. The RNA binding protein, fused protein in Sarcoma (FUS) [25,26], has a role in splicing regulation by means of alteration of FUS nuclear levels, while adenosine deaminases acting on RNA (ADAR) are known to bind double-stranded RNAs formed by IRAlus elements for adenosine to inosine (A-to-I) RNA editing [21,27], and heterogeneous nuclear ribonucleoproteins (hnRNPs) can bind to specific motifs in the flanking sites in order to form a looped structure [28].

In circRNAs flanking introns, reverse complementary match (RCM) is found to be contained in order to directly facilitate both exon-skipping and back-splicing, which is a conserved feature of circRNA biogenesis [22,29]. It has been established that RCM disruption can effectively abolish circRNAs formation [29]. In this way, the Alu elements repeated sequences could be manipulated to inhibit the formation of circRNAs. For example, where two Alu elements were found in the flanking introns of circRNA-0001875, a transcription factor SP1 could inhibit the circ-0001875 cyclization by binding to the upstream AluSq element and inhibiting the proximity of the splicing site [20].

QKI has been identified as interacting with the specific QKI response elements (QREs) in SLC26A4 gene introns, thereby promoting the biogenesis of circSLC26A4 [24]. FUS has been found to promote the splicing of circPDE4B [30]. Additionally, ADAR1 could promote circCHEK2 biogenesis by directly binding and editing the dsRNA structure formed in circCHEK2 flanking introns [21]. HnRNP-L, a multi-functional RBP, was also found to catalyze the high expression of circCSPP1 in prostate cancer [28]. In addition, the circRNAs biogenesis progression can be modulated by both intronic complementary sequences and RBPs in a combination manner. A recent study has identified that FUS and ADARB2 can act as vital regulators of circRHOBTB3 production in CRC cells by binding to specific motifs and Alu elements in the introns flanking the circRHOBTB3-forming exons [23].

## 3. The Classification of circRNAs

According to the components of the back-splicing junction site, circRNAs are commonly classified into three types: exonic circRNAs (EcirRNAs) [12], intronic circRNA (ciRNAs) [31], and exonic-intronic circRNAs (EIciRNAs) [32]. EcirRNAs and ciRNAs are circularized by exons and introns, respectively, while EIciRNAs are formed by exons with introns ‘retained’ between them. As a result, these different circRNAs can perform different functions.

Among these circRNAs, exonic RNAs are more prevalently located in the cytoplasmic cells, acting as miRNA sponges or decoys, protein sponges or decoys, protein scaffold, mRNAs regulator and so on. Both ciRNAs and EIciRNAs predominantly localize in the nucleus and are involved in local gene expression. They have been identified as positive regulators of RNA polymerase II transcription and as playing a crucial role in the efficient expression of their parental genes via specific RNA-RNA interaction in human cells, but they have little enrichment for miRNA target sites [31,32]. Currently, three hypothetical models of circRNAs formation are proposed to explain the back-splicing process: lariat-driven circularization, intron-pairing-driven circularization, and RNA-binding protein (RBP)-driven circularization [12,33]. In the lariat-driven circularization model, it has been reported that exon skipping is one of the factors responsible for the formation of circRNAs. During the RNA splicing process, linear mRNAs lacking of exons are produced while the skipped exons are contained in intron lariats, which are subsequently back-spliced to form mature circRNAs, joining the splice sites into close proximity [33]. As for the intron-pairing-driven and RBP-driven circularization, base pairing between repeats in flanking introns and RBPs, predominantly participate in the production of circRNAs.

## 4. The Detection Technology of circRNAs

The biological functions of circRNAs are largely determined by the sequence characteristics of circRNAs, such as encoding peptides, regulating parental gene transcription and so on [33,34]. In aim of exploring more landscape and function of circRNAs, there are constant searches for novel detection techniques for circRNAs. Along with the development of deep sequencing and ever-updating computational technology, a series of circRNAs detection methods have been developed to delineate the universal functional involvements of endogenous circRNAs, which have attracted more and more attention [35].

Traditionally, by using a combination of high-throughput sequencing or microarray analysis and quantitative real-time PCR, the differential expression of circRNAs in certain diseases can be screened, given the transcription’s abundant information of circRNAs, which always show several fold changes compared with the healthy control group [36]. The three-prime exonuclease RNase R treatment and addition of poly-A tails are often used in the RNA-seq samples to identify circRNAs of low abundance [37,38]. To fully explore the circRNAs’ landscapes, increasing lines of evidence show that many computational pipelines are applied in the accurate annotation and quantification of circRNAs from RNA-seq data [39,40,41]. For instance, to achieve more comprehensive quantification, a new integrative approach named Short Read circRNA Pipeline (SRCP) was used to validate and quantify circRNAs with high sensitivity and a low number of false negatives [42]. However, some widely used detection methods, such as Illumina sequencing, find it relatively hard to accurately quantify circRNAs due to the relatively short readings length [43].

Therefore, improved experimental strategies and computational tools for the accurate identification of the full length of circRNAs are needed. Recently, many full-length circRNAs sequencing methods such as isoCirc [44], CIRI-long [45], circNick-LRS [46], circFL-seq [47] have been developed to profile circRNAs at the isoform level. IsoCirc utilizes the rolling loop amplification and Oxford Nanopore sequencing to detect the full length of circRNAs and their internal splicing structures, overcoming the defect of short-read RNA sequencing [44]. Using CIRI-long, the full-length circRNAs can be reconstructed using nanopore sequencing, and the majority of identified circRNAs (96.57%) can be accurately determined at the BSJ site [45]. CircNick-LRS allows a detailed characterization of full-length circRNAs in all size ranges, identifying and quantifying specific splicing events in circRNAs. This long-read sequencing method can not only detect the internal composition of full-length circRNAs, including exon composition, intron retention, and microexons, but could also discover a series of circRNAs that have never been found using next-generation sequencing (NGS) [46]. CircFL-seq can detect the full length of circRNAs by rolling circular reverse transcription (RCRT) and nanopore long-read sequencing, which makes circRNAs reads more than ten-fold enriched compared to short-read RNA-seq. Moreover, as opposed to isoCirc and CIRI-long, circFL-seq adds poly(A) tail after RCRT and then the circRNAs sequences are amplified by adding primer connectors and captured variable isomers accurately, which provides it with the ability to identify more variable splicing events [47]. However, limitations on these emerging techniques should be acknowledged. In order to enrich circRNAs, IsoCirc and CIRI-long will carry out rRNA removal, RNase R enzyme treatment, and removal of RNA containing poly(A) tail on total RNA, leading to the comprehensive capture of linear and circRNAs. Simultaneously, some circRNAs can also be digested by RNase R enzyme, which causes the circRNAs’ library to deviate from the fact. Another problem is that the sequencing depth of IsoCirc and CIRI-long is not enough, and the detection rate of circRNA will continue to rise with deeper sequencing [44,45,46,47].

Furthermore, the association between circRNAs and diseases has captured the interest of researchers. Many computational methods can be used for predictions relevant to circRNAs-diseases association, such as iCircDA-MF [48], iCDA-CGR [49], iCDA-CMG [50] and GATNNCDA [51]. A computational method named KATZCPDA was applied to predict correlations between the circRNAs and diseases including tuberculosis and different kinds of cancers, such as colon cancer, glioma and breast cancer. As a result, it revealed that KATZCPDA had better predictive performance in verifying the existing circRNA-disease associations, as well as some unknown integrations among circRNAs, proteins, and diseases, which might play an essential role in the identification of potential circRNA-disease associations in the future [52].

Taken together, based on the rolling circles and long-read sequencing, accurate identification and quantification of full-length circRNAs introduce potentials for large-scale screening of functional circRNAs, while the development of prediction strategies to determine the association between circRNAs and diseases might be beneficial to disease prevention, diagnosis, and treatment, thereby outlining the horizon of the constant exploration for circRNAs.

## 5. Biological Roles of circRNAs

Along with the development of sequencing methods, a quantity of extant circRNAs has been identified, which brings mounting attention to the biological function of circRNAs in different biological responses. There is accumulating evidence to indicate that circRNAs exert many important biological effects, and the dysregulation of circRNAs has been associated with diverse pathological conditions, including Alzheimer’s disease (AD) [53], Severe Acute Respiratory Syndrome Coronavirus 2 (SARS-CoV-2) [54], Parkinson’s disease (PD) [55], acute ischemic stroke [56] and diverse cancers [24,57].

**Figure 1:**
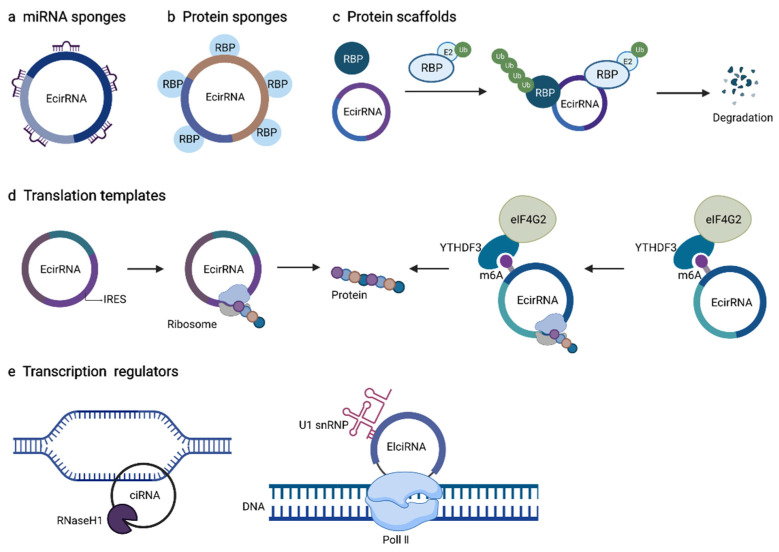
Schematic representations of several biological roles of circRNAs discussed in this article, including exonic circRNAs (EcirRNAs), intronic circRNA (ciRNAs) and exonic-intronic circRNAs (EIciRNAs). (**a**) CircRNAs can function as microRNA (miRNA) sponges or decoys by binding to the miRNA recognition elements (MREs) of miRNAs, thus affecting the expression of miRNA to target mRNA functions. (**b**) CircRNAs can function as key regulators of protein sponges or decoys by competing for the binding to the shared RNA binding protein (RBPs) binding motifs with specific RBPs. (**c**) CircRNAs are able to come into contact with two RBPs, and one of the proteins is often an enzyme such as E3 ubiquitin ligase, that is involved in the ubiquitination progress, which could lead to protein destabilization and degradation. (**d**) CircRNAs with internal ribosome entry site (IRES) elements can be translated to unique peptides. Also, circRNAs contain extensive N6-methyladenosines (m6A) modifications. For example, YTHDF3 as the m6A reader and eIF4G2 as translation initiation factor are sufficient to drive protein translation in a cap-independent fashion. (**e**) CircRNAs located in the nucleus can participate in regulating the expression of parental genes. For instance, circRNAs can interact with U1 snRNP and further bind to RNA polymerase II (Pol II) in the promoter region of gene to enhance the expression of parental genes. Moreover, ciRNAs can not only form a more stable R-loop with the parent gene DNA in a structure-dependent manner, but also can be degraded by RNase H1 in an R-loop-dependent manner, thus achieving transcription extension of parent genes.

Due to the important role of circRNAs, levels of circRNAs have been found to be related to overall survival [58], poor prognosis [57], and clinical stages [59,60], as well as differentiation of cancers [61]. As circRNAs show tissue-specific and disease-associated expression [62], more and more research efforts are trying to reveal the mechanisms of circRNAs regulated disease processes, which are related to the location of circRNAs in cells. In disease conditions, with an abnormal balance of subcellular localization of circRNAs, circRNAs may translocate within different parts of cells to exert their functions, which might trigger dysregulated signals in cellular processes [63,64]. CircRNAs have been shown to play important roles by acting as miRNA sponges, protein sponges or scaffolds, by regulating alternative RNA splicing or by being translated themselves (Figure 1).

### 5.1. Role of circRNAs as miRNAs Sponges or Decoys

Consisting of 19-22 nucleotides, miRNAs can bind to downstream target mRNA molecules, thereby leading to the destruction or translation suppression of target genes [65,66]. RNA transcripts often compete for binding to shared miRNAs, which prevents the miRNAs from inhibiting other mRNA targets [66]. Competitive endogenous RNAs (ceRNAs) is the term to describe the competition between different mRNAs, where these ceRNAs share miRNA recognition elements (MREs) to regulate each other [66,67]. Accumulating evidence has reinforced the perception that circRNAs could also competitively bind to the shared MREs of miRNAs, and thus sponge the target miRNA without affecting the expression of miRNA, while influencing downstream mRNA functions [68]. Up to now, these circRNA-miRNA-mRNA regulatory networks have been continuously delineated; the changes of circRNA-miRNA-mRNA regulatory networks might lead to significant insights into the mechanisms of circRNAs in human diseases.

It is now a widely accepted hypothesis that circRNAs exert functions similar to decoys of miRNA, a capacity also termed miRNA sponges, which refers to circRNAs with a long half-life that can bind to specific miRNAs through base-pairing and form complexes by hybridization. For example, a kind of circRNA highly expressed in the human and the mouse brain was proved to act as a miR-7 sponge or decoy, which was termed a transcript ciRS-7 [68]. The circRNA was completely resistant to miRNA-mediated target destabilization, which strongly suppressed miR-7 activity, resulting in increased levels of miR-7 targets. The AGO proteins are core components of the RNA-induced silencing complex, which are requisite for miRNAs to recognize their target genes and also an indication of the sponge potential of circRNAs [69]. Due to this regulatory pattern, RNA binding protein immunoprecipitation (RIP) assay and pull-down assay are often performed to confirm the interaction between circRNAs and the AGO2 protein. RIP assay mainly uses antibody for the target protein to precipitate the RNA-protein complex, and then verifies the RNA of the complex. As for pull-down assay, biotin-labeled RNA probes are used to pull down the RNA-protein complex and for further protein verification [70]. For instance, the level of testis-specific Sex-determining region Y (*Sry*) circRNA was found to be higher in the immunoprecipitation of AGO2 from miR-138-transfected cells compared to other miRNAs transfected cells. Transfecting miR-138 into the cell with the *Sry* expression vector, *Sry* circRNA was also specifically captured, which proved that the circular *Sry* RNA was able to efficiently sponge miR-138 [68].

CircRNAs harbor multiple numbers of binding sites to the same miRNA to act as a “sponge” and inhibit activity of miRNAs. It has previously been proposed that ciRS-7 contains more than seventy conserved target sites for miR-7 [68], and circSLC8A1 carries only seven binding sites for miR-128 [55]. In addition, individual circRNA can also bind to numerous miRNAs. For instance, circHIPK3 has been observed to sponge nine miRNAs with eighteen potential binding sites [71]. This function is also presented in different diseases; ciRS-7 was observed to strongly suppress miR-7 activity in neuronal tissues [68], however, it could also act as a sponge of miR-139-3p in renal cell carcinoma (RCC) to target TAGLN, which activated the PI3K/AKT signaling pathway and promoted cell proliferation, migration and invasion [72].

By repressing the expression of miRNAs, circRNAs were revealed as participants in the regulation of some important cellular signaling events, such as proliferation [71], migration [73], invasion [57,72], epithelial-mesenchymal transition (EMT) [59], apoptosis [74] and autophagy [75], thus contributing to the development of diverse diseases [24].

### 5.2. Role of circRNAs Contacting with RNA Binding Protein (RBPs)

RNA binding protein (RBPs) are a distinct class of proteins, predominantly regulating multiple post-transcriptional events in eukaryotic cells, including RNA stability, translocation, protein modification and translation [67]. It has been identified that circRNAs can interact with RBPs in a cell-type-specific manner via specific RNA-binding domains (RBDs), such as RNA recognition motifs (RRM), K homology (KH) domain and so on [58].

The physical interactions between circRNAs and RBPs play important roles under different circumstances. Individual circRNAs have the potential to interact with a single RBP or a class of different RBPs in diverse biological processes, while the same RBPs can also interact with several different circRNAs. For example, insulin-like growth factor 2 binding protein 3 (IGF2BP3) was experimentally shown to bind with circNEIL3 through the region of KH3–4 of in glioma cells [76]. Additionally, the interactions between Hsa_circ_0003258 and IGF2BP3 could promote prostate cancer metastasis [77] and circ-0039411 could recruit IGF2BP3 to promote malignant behaviors of LUAD cells [78].

CircRNAs display several roles during the interactions with RBPs to exert multifaceted effects on biological regulation, including translocation, mRNA stability and so on. Firstly, circRNAs can combine with RBPs for protein degradation of RBPs through ubiquitination degradation pathway, in which RBPs serving as ubiquitin binding enzyme E2. It has been reported that overexpressing of circRHOBTB3 facilitated the interactions between HuR and β-Trcp1. Therefore, circRHOBTB3 could bind HuR to promote E3 ubiquitin ligase β-Trcp1-mediated ubiquitination of HuR, exerting its effects by destabilizing HuR to regulate the levels of PTBP1-induced genes involved in cancer metastasis [23]. Secondly, circRNAs can come into contact with RBPs by competing with other proteins that bind to the same RBPs. For instance, the high expression of circNEIL3 in glioma cells was found to block the binding between IGF2BP3 and HECTD4, via the modulation of protein ubiquitination and degradation, as well as cellular immunosuppressive responses, thereby promoting malignant progression of glioma [76]. Thirdly, the interactions between circRNAs and RBPs can also influence the distributions of circRNAs. It has been demonstrated that circStag1 can interact with HuR and promote the translocation of HuR into the cytoplasm to activate the Wnt signaling pathway, which stimulates the osteogenic differentiation of BMSCs and bone regeneration [79]. Additionally, circCwc27 directly binds to purine-rich element-binding protein A (Pur-α), influencing its distribution by trapping Pur-α in the cytoplasm and then regulating Alzheimer’s disease genes transcription [53].

### 5.3. Role of circRNAs as Protein Sponges or Decoys

CircRNAs can function as key regulators of protein sponges or decoys by competing for the binding to the shared RBP binding motifs with specific RBPs. It was reported that exosomal circLPAR1 could serve as a sponge of eIF3h to inhibit the METTL3–eIF3h interaction and affect BRD4 protein expression, thereby suppressing colorectal cancer development [80]. In addition, circRNAs can also serve as a protein decoy to influence related mRNA stability. For example, circ-TNPO3 can interact with IGF2BP3 to weaken the role of IGF2BP3 in stabilizing MYC mRNA, thereby regulating the MYC-SNAIL axis and inhibiting the proliferation and metastasis of gastric cancer (GC) [61]. Another study identified that hsa_circ_0068631 could maintain c-Myc mRNA stability through recruiting EIF4A3, and then promoting breast cancer progression [81].

### 5.4. Role of circRNAs as Protein Scaffolds

Regarding the role as protein scaffolds, circRNAs are able to mediate the interaction between two proteins or mRNAs, and then form a RNA-protein ternary complex, exerting their stabilization or destabilization function [70]. It was identified that highly expressed circ-Foxo3 could interact with both cell cycle proteins CDK2 and p21 in non-malignant cell lines. The formation of this circ-Foxo3/p21/CDK2 ternary complex arrested the function of CDK2 and repressed cell cycle progression at the initiation of the transition from G1 to S phase [82].

Firstly, circRNAs function as a scaffold to enhance the interaction between two molecules to maintain the stability, which are beneficial for the disease’s progression. For example, circDCUN1D4 can strengthen the interaction between HuR protein and TXNIP mRNA to maintain the stability of TXNIP mRNA, which are involved in the metastasis and glycolysis of lung adenocarcinoma (LUAD) [73]. Secondly, circRNAs come into contact with two RBPs, and one of the proteins is often an enzyme such as E3 ubiquitin ligase that is involved in the ubiquitination progress, which could lead to the protein destabilization in the regulation of biological processes. One example of this is that circNDUFB2 can function as a scaffold to enhance the binding between TRIM25 and IGF2BPs and then facilitate the ubiquitination level of IGF2BPs, participating in the activation of anti-tumor immunity via RIG-I-MAVS signaling cascades during non-small cell lung cancer (NSCLC) progression [83].

### 5.5. Role of circRNAs as Templates for Translation

CircRNAs are traditionally classified as members of the non-coding protein RNAs owing to their lack of five-prime caps and three-prime tails which are required in the cap-dependent translation assay [84]. However, along with the high-throughput sequencing applied, emerging studies have identified that circRNAs harbor the protein-coding ability and can facilitate protein translation [85,86], especially the intronic RNAs (ciRNAs) that are predominantly detected in the nucleus [31]. Unlike the conventional translation in eukaryotes, circRNAs can function as a translator in a five-prime caps-independent translation method. Internal ribosome entry site (IRES)- and N6-methyladenosines (m6A)-mediated cap-independent translation initiation have been suggested to be potential mechanisms for circRNAs translation [87].

There are two fundamental factors that are vital for translation: translation initiation elements and potential open reading frame (ORF) [88]. IRES with 18S rRNA complementarity and a positioned stem-loop structured RNA element are required for driving circRNA translation, which can directly recruit and bind to ribosomes [89]. As the sequences that have the potential to encode proteins, ORF can work with regulatory elements such as IRES for translation. Translated circRNAs usually comprise the initiation codon AUG and putative ORF, and can efficiently translate proteins by a rolling circle amplification (RCA) mechanism. They continue to roll and pass through the back-splicing junction until they recognize the stop codon, which defines the success of translation or not [89,90]. However, the RCA mechanism can be used by the programmed−1 ribosomal frameshifting (-1PRF) to induce the break-up of out-of-frame stop codon (OSC). Afterwards, an endogenous rolling-translated protein is produced [91].

One representative protein-coding circRNA driven by IRES is circ-ZNF609, which contains an open reading frame and can be translated into a protein in a splicing dependent/cap-independent manner for the regulation of myoblast proliferation [92]. Similarly, circ-EIF6 could encode EIF6-224 aa, and circ-FBXW7 could encode FBXW7-185aa, to regulate TNBC progression [86] and glioma tumorigenesis [93], respectively. Moreover, in a recent research, extensive IRES-ribosome association was demonstrated and more than 17,000 endogenous and synthetic sequences were identified as candidate circRNAs IRES, most of which were located near the back-splicing junction of circRNAs [89], revealing that the translation of circRNAs was widespread.

Cells can use different translation machinery to respond to diverse conditions. Another translation method is m6A modification, which is widely accepted to be an important base modification pathway for RNA. Three elements, including readers, writers and erasers are required during this process. The m6A readers such as m6A binding protein can function on RNA, and writers such as methyltransferase and erasers such as demethylase are involved in the dynamic regulation of m6A in the nucleus [94]. It has been reported that circRNAs contain extensive m6A modifications and are sufficient to drive protein translation in a cap-independent fashion. The m6A-driven translation of circRNAs requires the m6A reader YTHDF3 and the translation initiation factors eIF4G2 and eIF3A, which is enhanced by methyltransferase METTL3/14, inhibited by demethylase FTO, and upregulated upon heat shock [95].

### 5.6. Role of circRNAs Function in Transcription

CircRNAs involved in gene expression regulation are mostly located in the nucleus, and the main ways of regulating parental gene expression can be listed as: protein modification, combining with parental genes, recruiting proteins to specific cell locations and so on [34]. A handful studies have revealed that circRNAs could influence transcript expression and function by interacting with RBPs involved in mRNA maturation [96]. For example, circPOK can bind to ILF2/ILF3, while ILF2/3 can further bind to the promoter region of IL-6. Additionally, during these processes, circPOK can enhance the activity of ILF2/3 in regulating IL-6 transcription and stability [97].

In addition, circMRPS35 located in the nucleus can recruit and bind with histone acetyltransferase KAT7 to induce histone acetylation of FoxO1/3a promoter region, which dramatically activated transcription of FOXO1/3a in gastric cancer progression [98]. Antisense circSCRIB can promote breast cancer progression by inhibiting parental gene splicing and translation [99]. CircSMARCA5 can inhibit the expression of the parent gene SMARCA5 by forming a secondary structure similar to R-loop and interact with the exon DNA of SMARCA5 to inhibit the DNA damage impairment process [34].

Circular intronic RNAs (ciRNAs) generated by co-transcription from pre-mRNA splicing is richly distributed in the nucleus and participates in regulating the expression of parental genes [31]. For example, Ci-ankrd52 can form a more stable R-loop with the parent gene DNA in a structure-dependent manner, which can not only replace the pre-mRNA chain from the previous R-loop, but also can be degraded by RNase H1 in an R-loop-dependent manner, thus achieving transcription extension of parent genes [100].

Li et al. first identified circRNAs that contained introns, ElciRNAs, which can interact with U1 snRNP to promote the transcription of parental genes. Some of these circRNAs can regulate the RNA polymerase II (Pol II) transcription of their parental genes in *cis* via forming specific RNA-RNA interactions. Using RNase H–based antisense oligonucleotides (ASOs) or short interfering RNAs (siRNAs) to deplete them could decrease the transcription levels of the corresponding parental genes. EIciEIF3J and EIciPAIP2 were taken as an example to identify the relationship between their linear forms. They were found to colocalize with their linear form gene, but not with the flanking genes. In addition, they were found to bind with U1 snRNP, and further bind to Pol II in the promoter region of EIF3/PAPI2 gene to enhance the expression of parental genes [32].

## 6. Potentials of circRNAs as Biomarkers in TB

At present, laboratory diagnosis of TB is mainly based on sputum specimen smear microscopy and sputum culture. However, the sputum specimen smear microscopy is limited by the low sensitivity and specificity values and the sputum culture is restricted by its long detection time, which may miss the proper treatment in time [101]. Therefore, it is of great significance to develop novel strategies for early diagnosis and timely treatments, thus curbing the spread of TB disease. Discovering molecular markers with significant distinguishing efficiency to discriminate between TB cases and healthy individuals are of vital importance to develop novel strategy for rapid diagnosis of TB.

It has been established that circRNAs are widely involved in a variety of physiological and pathological processes. The aberrantly expressed circRNAs found in patients might hint at the potential role of circRNAs in the diagnosis of diverse diseases [102]. As for the wide distribution and stability characteristics, circRNAs are able to be easily detected in body fluids, such as blood, urine, exosomes and so on [103,104]. Recently, more and more studies are suggesting that circRNAs can act as diagnostic biomarkers for TB [105,106,107,108,109,110,111,112,113]. We have summarized of all the circular RNAs that are currently reported to be involved in regulating Tuberculosis in Table 1. However, there are still many aspects of the question that have yet to be sufficiently explored to learn about the essential roles of circRNAs after tuberculosis infection, which might enhance our understanding of TB pathogenesis and benefit the development of TB diagnostic or therapeutic strategies.

Regarding the feasibility application of circRNAs as potential biomarkers for TB diagnosis, the method of identifying TB-related functional circRNAs is a critical issue. The circRNAs microarrays can be used to screen the circRNAs expression profiles between Mtb-infected group and control group. Then differentially expressed circRNAs can be verified by quantitative real-time polymerase chain reaction (qRT-PCR). Additionally, receiver operating characteristic curve (ROC) or the area under the ROC curve (AUC) can be applied to evaluate the diagnostic value of circRNAs for TB with the sensitivity and specificity values determined based on the respective cut-off values [106].

In term of circulating circRNAs as potential biomarkers, there are three perspectives related to the diagnosis and progression of TB. Firstly, emerging studies have identified that various kinds of samples in TB patients, including plasma, serum, PBMCs, MDMs, whole blood and exosomes, contain diverse levels of dysregulated circRNAs for potential TB diagnosis. The alterations of circRNAs are involved in human immunological responses against TB infection. Plasma level of hsa_circ_103571, human MDMs level of hsa_circ_0043497 and hsa_circ_0001204, and serum levels of circRNA_051239, circRNA_029965, and circRNA_404022 in active TB samples were found to be served as potential biomarkers for TB diagnosis [106,107,108]. Another study first analyzed the expression profile of circRNAs in PBMCs from three active pulmonary tuberculosis (APTB) patients and three healthy controls by microarray screening. Six circRNAs were then chosen for validation using qRT-PCR in 40 TB patients and 40 control subjects. The AUC of six candidate circRNAs were all larger than 0.750, suggesting their potential diagnostic value in TB diagnosis. In order to further verify the specificity of selected circRNAs and their abilities to effectively distinguish TB from other lung diseases according to the circRNAs expression levels, further evaluation was performed in an independent cohort consisting of 115 TB patients, 40 pneumonia patients, 40 COPD patients, 40 lung cancer patients and 90 control subjects. It was established that the expression of hsa_circRNA_001937 is significantly higher in TB patients compared with that in patients with other pulmonary diseases such as pneumonia, COPD and lung cancer, revealing that hsa_circRNA_001937 may serve as a TB-specific signature circRNA and could be used as a candidate biomarker of TB [111].

Our previous work also focused on six differentially expressed circRNAs in the PBMCs of APTB through high-throughput sequencing validated in 10 APTB patients and ten health volunteers. Furthermore, the verification of hsa_circ_0005836 and hsa_circ_0009128 was also conducted between 34 APTB and 30 health controls. It has been demonstrated that hsa_circ_0005836 and hsa_circ_0009128 expression levels were significantly down-regulated in the PBMCs of APTB, which indicated their potentials as new diagnostic biomarkers and therapeutic target of active TB [105]. By comparing the peripheral blood of 31 healthy controls and 32 active TB patients, hsa_circ_0001380 was found to be down-regulated in TB patients with a diagnostic value evaluated by AUC of 0.9502. These results indicated high sensitivity and specificity of 93.75% and 87.50%, respectively, suggesting the high diagnostic value of hsa_circ_0001380 in APTB [114].

Moreover, the combination of several circRNAs as biomarkers can increase the diagnostic value. For instance, hsa_circRNA_001937 showed 85% of sensitivity and 77.5% of specificity as candidate biomarkers for TB diagnosis, while hsa_circRNA_009024 showed 75.0% of sensitivity and 80.0% of specificity. Importantly, the sensitivity and specificity for the combination of hsa_circRNA_001937 and hsa_circRNA_009024 are found to reach 95.0% and 80.0%, respectively, which are higher than that of single circRNA and can provide better diagnostic accuracy with the AUC of 0.926 [111]. In addition, in a completely different sample comprising 120 TB and 100 healthy control subjects, the diagnostic potential of combining hsa_circ_0001953 and hsa_circ_0009024 in the clinical setting was determined. The AUC, sensitivity and specificity for hsa_circ_0001953 were 0.826, 69.17% and 89.00%, respectively. For hsa_circ_0009024, an AUC of 0.777 was obtained, and sensitivity and specificity were 60.00 and 86.00%, respectively. Notably, when the plasma levels of hsa_circ_0001953 and hsa_circ_0009024 were used in combination, the AUC of 0.915 was obtained for detecting TB, with sensitivity and specificity of 72.50% and 96.00%, respectively [109]. Also, plasma levels of hsa_circ_0001204 and hsa_circ_0001747 were selected for further analysis in 145 TB patients and 120 control individuals, which then showed AUC of 0.871 and 0.830, respectively. However, the AUC for distinguishing TB patients was increased to 0.928 (sensitivity = 86.21%, specificity = 89.17%) when hsa_circ_0001204 and hsa_circ_0001747 were used in combination. Recently, a study determined the ncRNAs expression profiles in exosomes derived from H37Ra and BCG infected macrophages. A total number of 2332 circRNAs were recognized associated with TB infection and some circRNAs such as hsa_circ_0129477, hsa_circ_0082641, hsa_circ_0072892, hsa_circ_0104568 and hsa_circ_0036372 were predicted to interact with known miRNAs, which revealed the potential functional circRNAs markers in the pathogenesis and diagnosis of TB [122].

Secondly, circRNAs are able to evaluate the clinical curative effect in TB. It has been identified that the abnormal plasma level of circRNAs can be markedly altered to the normal level in TB patients upon successful therapy. For instance, hsa_circRNA_001937 expression is elevated in active TB patients, which is significantly reduced to the normal level after successful anti-TB therapy through eight-month following up in 20 newly diagnosed TB patients [111]. Additionally, the expression of hsa_circ_0001204 in plasma and MDMs both decreased in TB patients, and hsa_circ_0001204 expression can be significantly up-regulated after anti-TB treatment [107,110]. Hsa_circ_0001953 and hsa_circ_0009024 expression levels are markedly decreased in TB patients upon successful therapy; this was assessed in 25 TB cases pre- and post-treatment, with no significant difference between the control and TB treated group [109]. Additionally, as well as distinguishing TB patients and health controls, circRNAs also showed strong potential for diagnosis of drug-resistant TB. In a recent study, the plasma levels of circRNAs were analyzed in 20 drug-resistant TB patients and 31 pan-susceptible TB patients, and found that circRNA_051239 was significantly increased in the drug-resistant group, revealing a marker that could be used to distinguish drug-resistant TB patients from pan-susceptible TB patients [108]. Additionally, in a study related to the anti-TB drug-induced liver injury (ADLI) in TB patients, peripheral blood from sixteen ADLI patients and 16 non-ADLI patients were collected and isolated to obtain total RNAs for human circRNAs microarray expression profiling. Among the ADLI-specific circRNAs, the expression of circMARS was found to be elevated in the serum of TB patients, which was validated by qRT-PCR in a cohort consisting of 150 ADLI patients and 150 non-ADLI patients. Also, cytology experiments and a self-controlled cohort of 35 participants before and during ADLI were used to verify the function of circMARS after anti-TB treatment. In this way, circMARS could act as a miR-6808-5p/-6874-3p/-3157-5p sponge to participate in the compensatory repair of ADLI, which might hamper the achievement of the treatment goals of TB [123].

Thirdly, circRNAs harbor the potential to distinguish the degree of TB disease or TB disease progress. Some circRNAs are reported to be positively correlated with TB severity. For instance, in clinical settings, according to pulmonary radiographic images, APTB patients can be classified into three severity levels: minimal, moderate and advanced disease. By using a double-blind test to classify 40 APTB patients regarding the severity of disease, each one gained radiological severity scores (RSS), and the number of minimal, moderate and advanced disease were 18, 12 and 10, respectively. Then the correlation between circRNAs levels and RSS were analyzed by using Spearman’s rank correlation test, and three of the six differentially expressed circRNAs were found to be correlated with the RSS, including hsa_circRNA_001937, hsa_circRNA_009024 and hsa_circRNA_102101 [111]. Similarly, hsa_circ_0001204 and hsa_circ_0001747 were found to be markedly down-regulated, while hsa_circ_0001953 and hsa_circ_0009024 were up-regulated in the plasma levels of active pulmonary TB patients [109,110]. Both of their plasma levels are correlated with the radiological severity scores, which are assessed according to pulmonary radiographic images, implying that these circRNAs might be involved in the disease progress of TB and associated with TB pathology.

Also, Lyu et al. have summarized and validated the TB-related circRNAs diagnostic panels to reveal their performance and biological function in datasets. They indicated that the parental genes of circRNAs mostly participated in the biological processes including GTPase activity and protein autophosphorylation, while circRNAs in related panels were involved in Wnt and JAK-STAT signaling pathways through GO and KEGG analysis [124]. This work provides a potential basis for clinical choice of TB-related circRNAs diagnostic panels. There is no doubt that the circRNAs chosen as TB-markers can be somehow involved in different pathways, which might present a puzzle in diagnosing a healthy patient with false positive signals. It is still necessary to combine other clinical detection for TB diagnosis such as Ziehl-Neelsen acid fast staining analysis of the sputum smears and lung images to eliminate the interference signals from other diseases [105,111].

## 7. CircRNAs Regulate Anti-TB Defense as Potential Therapeutic Targets

Upon Mtb infection, host macrophages are activated as the first line of defense to fight against the bacteria. Intriguingly, Mtb can successfully evolve into several mechanisms to escape from bactericidal activities of macrophages, thus safely surviving inside the cell host, and leading to a life-long latent infection or even active TB [125]. An increasing number of studies have indicated that circRNAs can act as the key molecules involved in the immune defense response during TB infection through modulating macrophages’ functions, with the aim of controlling and eliminating of Mtb invaded in macrophages [118,119,120]. Therefore, it is of great significance to identify the relevance of aberrantly expressed circRNAs in TB and to clarify their potential mechanisms involved in the pathogenesis of TB infection.

Previous studies have identified the potential interactions for circRNA-miRNA-mRNA in TB, which might provide some detailed mechanisms for TB development in genetic scale. By circRNA/miRNA interaction prediction, Fu et al. found that circRNA_101128 expression in TB samples was negatively correlated with the level of its possible target let-7a and circRNA_101128 was potentially involved in MAPK and P13K-Akt pathway possibly via modulation of let-7a [112]. Luo et al. indicated that hsa_circ_0001380 in blood sample could be a biomarker for active pulmonary TB and the potential target miRNAs might be hsa-miR-622 and hsa-miR-136-5p [114]. Hsa_circRNA_103571 expression was significantly decreased in the plasma of active TB patients and showed potential interaction with active TB related miRNAs, such as miR-29a and miR-16 [106]. Additionally, circRNA_051239 might act as a ceRNA for miR-320a, and play a vital role in the TB drug-resistant progress [108]. Circ_0001490 expression was down-regulated both in the serum samples of TB patients and Mtb-infected THP-1 macrophages. Additionally, circ_0001490 could interact with miR-579-3p as miRNA sponge, and miR-579-3p could interact with the 3′ untranslated region (3′UTR) of follistatin-like protein 1 (FSTL1). Thus, overexpression of circ_0001490 suppressed Mtb survival and promoted the viability and inflammatory response of Mtb-infected THP-1 macrophages partly by regulating miR-579-3p/FSTL1 axis based on its miRNA sponge roles [120]. The expression of cPWWP2A was down-regulated in Mtb-infected macrophages and was identified to function as an endogenous miR-579 sponge, inhibiting miR-579 activity and expression. Conversely, ectopic cPWWP2A over-expression largely attenuated the cytotoxicity and apoptosis of macrophage induced by Mtb infection, suggesting that targeting this cPWWP2A/miR-579 axis might be a novel strategy to protect human macrophages from Mtb infection and provide new directions for possible clinical TB control [121]. These results indicate that circRNAs can function as miRNAs sponges or decoys to regulate different progress of TB.

Among the immune escape mechanisms exploited by Mtb, cellular apoptosis and autophagy exert essential roles in the control of bacterial infection [126]. As a part of innate immune response, apoptosis is regarded as an efficient strategy for intracellular pathogens removal, via potently eliminating their replicative niche, thus restricting the transmission of the infection [127]. It has been identified that Mtb can inhibit macrophage apoptosis, reversing the manipulation of host immunity. Unlike apoptosis, autophagy is shown to maintain the homeostasis in host cells without inducing cell death, which therefore can be developed to inhibit intracellular Mtb growth by inducing autophagy in macrophages [128].

Given these two major roles, the immunity status of macrophages is manipulated by Mtb in order to circumvent host immune response and promote its intracellular survival. For example, circAGFG1 was of the type of circRNAs to balance the physiological and pathological activation of macrophages caused by autophagy and apoptosis. CircAGFG1 was highly expressed in active TB and was sufficiently plastic to the modulation of Mtb by enhancing autophagy but reducing apoptosis via the miRNA-1257/Notch axis, which would benefit of the host defense against Mtb [119].

Moreover, many circRNAs induce autophagy in the progression of TB, suppressing the intracellular survival of Mtb. For example, hsa_circ_0045474 was down-regulated in monocytes from TB patients and induced macrophage autophagy, via modulation of miR-582-5p/ TNKS2 axis, implying a potential strategy to treat the occurrence of active pulmonary TB by targeting hsa_circ_0045474 [118]. According to the principle that the physiological activation of autophagy in macrophages could facilitate mycobacterial clearance, circTRAPPC6B-enhanced autophagy aggregation or sequestration have been observed for intracellular Mtb inhibition by our group [116]. The overexpression of circTRAPPC6B could enhance the colocalization of LC3B puncta and GFP expressing BCG in THP-1 macrophages, exhibiting elimination of invading pathogens. In detail, circTRAPPC6B could antagonize the ability of miR-874-3p to suppress ATG16L1 and enhance autophagy sequestration to restrict Mtb growth in macrophages.

Additionally, macrophages can differentiate into several specific phenotypes in response to various stimuli in diverse physiological and pathological contexts, thereby exhibiting various functionalities [129]. It is widely accepted that macrophages can be classified into two subtypes: classically activated M1 macrophages and alternately activated M2 macrophages. M1 macrophages can lead to pro-inflammatory responses with increased microbicidal capacity, while M2 macrophages can lead to anti-inflammatory responses with lower microbicidal capacity [130]. Previous studies have also identified that Mtb infection can induce the polarization of monocyte-derived macrophages [131]. Similarly, M1 macrophages could promote granuloma formation and macrophage bactericidal activity in vitro, while M2 macrophages inhibit these effects.

For example, hsa_circ_0003528 was found to be elevated in the plasma of active pulmonary TB patients in comparison with that of healthy controls. Additionally, has_circ_0003528 can function as ceRNAs (including sponging miR-224-5p, miR-324-5p and miR-488-5p), targeting the co-share gene CTLA4. This work demonstrated that up-regulation hashsa_circ_0003528 could promote TB associated macrophage polarization by up-regulating the expression of CTLA4, reminding us of the critical roles of circRNAs in macrophage polarization regulations against Mtb infection [117].

Relying on the high-throughput studies and bioinformatic analysis, an increasing number of circRNAs related to Mtb infection can be emergingly predicted and recognized. Based on circRNA-miRNA-mRNA and protein-protein interaction networks, three hub genes associated with the development of pulmonary TB were identified, including hsa_circ_0007919, hsa_circ_0002419, and hsa_circ_0005521. Such a prediction might provide references to find candidate biomarkers for the early diagnosis of pulmonary TB and for possible therapeutic strategy development [132]. By using prediction databases online, circRNAs such as SAMD8_hsa_circRNA994 and TWF1_hsa_circRNA9897 were found be significantly correlated with the interferon signaling pathway, as potential molecules to act as a defense against TB infection in host cells [115].

## 8. Conclusions and Prospective

In this review, we briefly summarize the background of tuberculosis and circRNAs, including the biogenesis and detection technology of circRNAs. Along with the aim of furthering the circRNAs progression in TB, we focus on the crucial immunity roles of circRNAs in regulation of tuberculosis: acting as miRNAs sponges/decoys or protein sponges/decoys, protein scaffold, transcription and translation. Moreover, through modulation of apoptosis, autophagy, inflammation and other pathways, circRNAs directly or indirectly affect the survival of Mtb, ultimately affecting occurrence of TB.

Compared with linear RNAs, circRNAs are highly stable and covalently closed loops which can protect them from exonuclease degradation. Owing to the ring-shaped stable structure and the wide distribution characteristics, up-regulated or down-regulated expression of circRNAs can be used as novel biomarkers in diverse diseases. The ubiquitous expression and high conservation of circRNAs are widely found during evolution and disease occurrence/progression. Therefore, circRNAs can be targeted for treatment and prognosis prediction of diseases, which might help to reveal the mechanisms and functions of various circRNAs in TB.

Regarding to the roles of circRNAs as biomarkers in TB diagnosis, several limitations in current studies should be acknowledged. Firstly, the relatively small sample size in the majority of independent studies provided limited information for deep understanding of the RNA functions and mechanisms. Further verification in larger and more diverse patients’ cohorts are required, as well as their long-term follow-up clinical information. Secondly, the different origins of samples make significant differences in the circRNAs expression profiling, which relies on the discussion in the same single-center studies in comparison. Thirdly, although circRNAs are taking part in the distinguishing of TB, the combinations with other detection methods, such as sputum specimen smear microscopy and pulmonary radiographic imaging, continue to be necessary for more accurate diagnosis.

To date, as to the mechanisms and functions of various circRNAs in TB, most of the studies have reported the involvement of circRNAs in TB through the miRNA-mRNA transcriptional regulatory axis, while their functions, such as interacting with RBPs, or participating in transcription or translation, have not been explored. In future research, taking the advantage of newly born technologies, it will be very necessary to unveil the roles and mechanisms of circRNAs in TB. Further development of a novel regulatory networks analysis strategy of circRNAs would also be helpful in exploring new functions and underlying mechanisms in TB, which would finally be beneficial and crucial for the prevention, control and treatment of TB.

Although advances in circRNAs research have enabled the elucidation of correlation in some diseases such as TB, gastric cancer [61], lung adenocarcinoma [73], and so on, there are thousands of circRNAs that are still unclear in the function perspective. Interestingly, circRNAs were once considered as “trash” sequences in the splicing process of mRNA, but now circRNAs have become important and are currently being studied extensively in almost all biological and biomedical fields. CircRNAs with clear functions can be used not only as biomarkers for the diagnosis, but also as therapeutic targets for some important diseases. Owing to the specific characteristic that circRNAs can translate proteins without the cap, applying circRNAs on RNA therapy might be a very promising idea. Recently, it has been reported that circRNAs opened up potential tools for developing highly effective vaccines against COVID-19 strains [133], suggesting a promising research avenue. Penultimately, the safety of circRNAs as novel tools still awaits further investigation. CircRNAs drugs targeting immune diseases or cancers can be prepared using circRNAs technology in vitro [134], and the immunogenicity and biosafety of circRNAs vaccines or drugs needs to be further confirmed in vivo and in vitro [135]. In conclusion, along with the further studies on the functions and mechanisms of circRNAs with the sequentially updated methods, we believe that circRNAs would emerge more and more as having significant roles in the control of TB.

## Figures and Tables

**Table 1:** Summary of all the circular RNAs that are currently reported to be involved in regulating Tuberculosis.

Circular RNA	Function	Expression	Derived From	Targets/Signaling Pathways	AUC	Number of TB Patients/Controls	Ref.
hsa_circ_0001204	biomarker	down	plasma		0.871	145/120	[110]
hsa_circ_0001747	biomarker	down	plasma		0.830	145/120	[110]
hsa_circ_0001204; hsa_circ_0001747	biomarker	down	plasma		0.928 *	145/120	[110]
hsa_circ_0001953	biomarker	up	plasma		0.826	120/100	[109]
hsa_circ_0009024	biomarker	up	plasma		0.777	120/100	[109]
hsa_circ_0001953; hsa_circ_0009024	biomarker	up	plasma		0.915 *	120/100	[109]
hsa_circ_001937	biomarker	up	PBMCs		0.873	115/90	[111]
hsa_circ_0043497	biomarker	up	Mtb-infected MDMs		0.860	96/85	[107]
hsa_circ_0001204	biomarker	down	Mtb-infected MDMs		0.848	96/85	[107]
hsa_circ_103017	biomarker	up	PBMCs		0.870	31/30	[112]
hsa_circ_059914	biomarker	up	PBMCs		0.821	31/30	[112]
hsa_circ_0028883	biomarker	up	PBMCs	miR-409-5p	0.773	20/20	[113]
hsa_circ_0005836	biomarker	down	PBMCs		no mention	49/45	[105]
hsa_circ_0001380	biomarker	down	PBMCs		0.9502	32/31	[114]
hsa_circ_103571	biomarker	down	plasma		0.838	32/29	[106]
circ_051239	biomarker	up	serum		0.9738	72/30	[108]
circ_029965	biomarker	up	serum		0.9443	72/30	[108]
circ_404022	biomarker	up	serum		0.9682	72/30	[108]
SAMD8_ hsa_circRNA994	no mention	no mention	whole blood		no mention	45/61	[115]
TWF1_ hsa_circRNA9897	no mention	no mention	whole blood		no mention	45/61	[115]
circTRAPPC6B	miRNA sponge	down	PBMCs	miR-874-3p ATG16L1 autophagy	0.8609	32/31	[116]
hsa_circ_0003528	miRNA sponge	up	plasma	miR-224-5p miR-324-5p miR-488-5p CTLA4 polarization	no mention	50/50	[117]
hsa_circ_101128	biomarker; miRNA sponge	up	PBMCs	let-7a MAPK/P13K-Akt pathway	0.817	31/30	[112]
hsa_circ_0045474	miRNA sponge	down	PBMCs	miR-582-5p TNKS2 autophagy	no mention	15/15	[118]
circAGFG1	miRNA sponge	up	alveolar macrophages in ATB patients	Notch miR-1257 apoptosis autophagy	no mention	no mention	[119]
circ_0001490	miRNA sponge	down	Mtb-infected THP-1 macrophages; serum	miR-579-3p FSTL1 inflammatory response	no mention	40/23	[120]
cPWWP2A	miRNA sponge	down	primary human MDMs	miR-579	no mention	no mention	[121]

* The AUC for distinguishing TB patients when several circRNAs are used in combination.
